# A Forgotten Medical Hero

**Published:** 1895-11-16

**Authors:** 


### A Forgotten Medical Hero.

Sir Richard Quain has a quarrel with English civilisation because it so often fails to single out for honour those to whom honour is due. Civilisation would do well if it would quarrel with itself on this account. There is nothing which makes so surely for true greatness in a nation as the putting of the worthiest in the worthiest place. The particular occasion which has evoked Sir Richard's protest is the absolute oblivion into which the name of the late Dr. Snow has fallen.

What does this country, and what, by consequence, does all civilisation owe to Dr. Snow? Our country owes to him, first and chiefly, its immunity from epidemic cholera during the past quarter of a century, and, of course, for all future time; and, as Sir Richard Quain expresses it, "millions" of us owe him our "lives." Yet so little is the name of this noble benefactor of mankind known, that probably not one in a dozen, even of medical men, under forty years of age, has so much as heard of him.

A few biographical words will put readers in possession of the main facts of Dr. Snow's work in as far as it concerns us here. Cholera, the scourge of the East, and which threatened to be the scourge of the West, was discovered by Dr. Snow, within the memory of men scarcely old, to be a waterborne disease. In that discovery lay the secret of its practical extirpation. To keep drinking water sweet is the salvation of the community from cholera; to avoid the drinking of any water which may be contaminated by the excreta of cholera patients, or by cholera germs from any other source, is the sure salvation of the individual, even in the midst of raging epidemics. This, as we have said, was the great discovery which crowned the researches of Dr. Snow. Every subsequent research has added proof upon proof to the validity of that conclusion. To-day the pregnant discovery is the common property of physicians and sanitarians in every part of the civilised world; and every intelligent man and woman, and every well-ordered state and city, now sleeps in peace, even when threatened by the approach of the deadly plague, because of the knowledge that prevention is entirely a matter of the supplying and drinking of uncontaminated water.

It might have been an accident which revealed this all-significant truth to Dr. Snow. Even if it had been, some honour should have been done to him, because its significance to the world would have been entirely the same. But it was no accident. On the contrary, it was only after long and patient research, and the addition of fact to fact and argument to argument, that he was able to demonstrate the irrefragable certainty of his great discovery. Men of the first eminence, such as Dr. Baly and the late Sir William Gull, for long maintained an uncompromising opposition; and it is probable that Snow, who died at the early age of fifty, hardly lived to see them all convinced.

Sir Richard Quain sums up his indictment against English civilisation in a sentence: "Dr. Snow made us masters of the deadly plague of cholera." Dr. Snow thereby saved millions of lives. The sole reward which England has conferred upon Dr. Snow is midnight oblivion. If Snow had been a soldier—Sir Richard Quain complains—if he had slain his thousands instead of saving his millions, every town would have hailed him a hero, and the nation would have honoured his memory with monuments more enduring than brass. No doubt that is so; and yet we cannot altogether blame the nation. The soldier is a very indispensable person, and though he slays rather than saves, he slays that his country may be saved, not only in its life, but in his freedom and its greatness.

If we were to probe the facts to the bottom we should probably find that professional jealousy had much to do with obscuring Snow's great work. The unscientific community could not take the true measure of his researches and conclusions. It was for his own profession to make that work clearly "understanded of the people." We have seen how Gull and Baly opposed. If they were sceptical, how could the majority of the profession believe? Above all, if medicine as a whole withheld its faith, how could the unscientific public be more appreciative than its medical guides? Upon medicine itself, we fear, must be placed the obloquy of the obscuring of a great medical name.

Medicine has often before made the *amende honorable*, especially after the prophet's death. It is promising, in the person of Sir Richard Quain, to medically canonise Snow. So let it be. It is better that we should honour a great name now, at any rate for the sake of our own honour and mental peace, than that we should not do it at all. "Before I leave this earth," said Sir Richard Quain, "I hope I shall secure something in the shape of a memorial to Snow." It was right well spoken, Sir Richard! All that is wanted is a beginning: a beginning by responsible persons. A drinking fountain on the Thames Embankment is the form of memorial proposed. The particular form is of little moment. What is wanted is that a memorial, a national memorial, or better still, a memorial by the whole of civilisation, shall be raised to the man who has made all civilisation his debtor for life and well-being. What the medical profession can do, and ought to do, is to make a beginning. If that beginning be generously made, and if the facts in their true light and their full significance be placed enthusiastically before the English-speaking world, we shall not have many years to wait for an adequate memorial to a great medical name. "Before he leaves the world" Sir Richard Quain may reasonably hope to see this honourable work well done.

